# Morphological description and molecular identification of *Myxobolus dajiangensis* n. sp. (Myxozoa: Myxobolidae) from the gill of *Cyprinus carpio* in southwest China

**DOI:** 10.7717/peerj.13023

**Published:** 2022-03-04

**Authors:** Miao-miao Wang, Jin-ye Zhang, Yuan-jun Zhao

**Affiliations:** Chongqing Key Laboratory of Animal Biology, College of Life Sciences, Chongqing Normal University, Chongqing, China

**Keywords:** *Myxobolus dajiangensis* n. sp., *Myxobolus koi*, Morphology, SSU rRNA gene, Cryptic species

## Abstract

**Background:**

Myxosporean diversity is a hot topic since they are difficult to accurately identify and classify. Many *Myxobolus* parasites have been named as *Myxobolus koi* because of their similar morphological features with the species originally reported. However, the distinctions in fine morphological features, host specificity, and molecular data have given rise to the attention of researchers.

**Methods:**

The classical morphometric and histological methods were used to describe the *Myxobolus dajiangensis* n. sp. in morphology. The common techniques in modern molecular biology and the methods of phylogenetic analyses were combined to identify the species.

**Results:**

Plasmodia of interlamellar-vascular type were found in the vascular network of gill lamellae. Mature myxospores of *M. dajiangensis* n. sp. were elongated and pyriform from the frontal view. The myxospores were 14.8 ± 0.4 (13.9–15.6) µm in length, 8.0 ± 0.5 (7.2–9.1) µm in width, and 5.5 µm in thickness. The two polar capsules were pyriform and slightly different in length. The length of the larger polar capsules was 8.0 ± 0.4 (7.1–8.8) µm, and it was 7.4 ± 0.4 (6.1–8.0) µm for the smaller ones. The width of both polar capsules was 2.5 ± 0.2 (2.0–3.2) µm. The polar filaments within the polar capsules were each coiled nine to 11 turns. Comparative analysis of both the morphological and molecular data between the present speices and other similar species revealed that the present species is a novel species, *Myxobolus dajiangensis* n. sp. Also, *M. koi* (FJ710800) was misidentified and the congener with *M. dajiangensis* n. sp., depending on the secondary structures of SSU rRNA and phylogenetic analysis. Moreover, the cryptic species existed in the *M. koi* parasites.

## Introduction

Since their discovery in the early 19th century, myxozoans have attracted great attention. To date, more than 2,600 myxozoan species, representing approximately 20% of cnidarians, have been described worldwide (Okamura, Hartigan & Naldoni, 2018; Eiras et al., 2021). Among them, over 900 nominal species belong to the genus *Myxobolus* Bütschli, 1882. They are diverse and widespread (Eiras et al., 2021). Although the taxonomic methods for myxozoans are improved continuously, it is still challenging to identify many species with similar morphology (Kaur & Singh, 2012), which leads to the mistake of species identification and the emergency of cryptic species, thus hiding the diversity of myxosporean to a certain extent (Eszterbauer, 2002; Bartošová-Sojková et al., 2014; Hartikainen et al., 2016; Liu, 2014; Liu, Yang & Zhao, 2016).

*Myxobolus koi* Kudo, 1919 is originally isolated from the connective tissue of the gill filament of *Cyprinus carpio* Linnaeus, 1758 in Japan and named (1920). Subsequently, a series of information about those parasites named *M. koi*, including the characteristics in morphology, geographical distribution, their hosts, pathogenicity, and therapeutic methods, have been taken notes successively by lots of scientists (Nakai, 1926; Hoshina, 1952; Akhmerov, 1960; Shul’man, 1966; Crawshaw & Sweeting, 1986; Chen & Ma, 1998; Rukyani, 1990; Yokoyama et al., 1997; Camus & Griffin, 2010; Liu, 2014). Moreover, the morphometric and molecular data, hosts, and localities are collected in Table 1, indicating that some data are not inconsistent between the *M. koi* originally reported and later published. Nakai (1926) has detailedly described the tissue parasitic pattern, plasmodium development, and spore morphological characteristics of a new species “so-called *Myxobolus koi*” with the same scientific name, which is also isolated from the same site of the same host. However, the two sets of morphometric data provided by both Kudo and Nakai are not overlapped. Furthermore, Egusa (1978, 1983) has researched all previous data and also pointed out the morphological differences between the *M. koi* originally reported by Kudo and published later by Nakai. To the best of our knowledge, the different species with quite a resemblance in morphology can parasitize the same site of the same host. For example, *Myxobolus paratoyamai* Kato et al., 2017, *Myxobolus toyamai* Kudo, 1917, *Myxobolus tanakai* Kato et al., 2017, *Myxobolus parakoi* Liu et al., 2019, and *M. koi* all parasitize the gill lamellae of *C. carpio* (Kato et al., 2017; Liu, Zhang & Zhao, 2019; Yokoyama & Ogawa, 2015; Zhang et al., 2020). Therefore, the species recorded independently by Kudo and Nakai are probably homonymous and heterogeneous despite their similarity in morphology and infecting the same site of the same host. In addition, the members of the genus *Myxobolus* are of host highly specificity, while the hosts of *M. koi* recorded by Akhmerov (1960), Shul’man (1966) and Chen & Ma (1998) are extensive (Table 1). Until 2009, Makoto and Hiroshi have uploaded the first SSU rDNA sequence (Accession No. FJ710800) to GenBank. So far, six sequences of SSU rDNA for *M. koi* have been submitted in GenBank, and one is provided by our research team. Based on morphological taxonomy, SSU rRNA secondary structure, and phylogenetic analysis of several known *M. koi* parasites, previous research has shown that *M. koi* (FJ710800) should be a different organism from the other *M. koi* species (Zhang et al., 2020). Therefore, the fact is inferred that two or more species may be assigned as *M. koi* because of various reasons, such as the incomplete description of original species in morphology and the limitation of research technologies.

**Table 1:** Morphometric comparison of the present species with morphologically similar species.

Species names	SL (μm)	SW (μm)	ST (μm)	PCL (μm)	PCW (μm)	PCT (μm)	NFC	Cyst (mm)	Host	Locality	Accession No.	Reference
*Myxobolusdajiangensis* n. sp.	14.8 ± 0.4 (13.9–15.6)	8.0 ± 0.4 (7.3–9.1)	5.5	7.8 ± 0.5 (6.1–8.8)	2.5 ± 0.2 (2.0–3.2)	4.0	9–11	0.2–0.8	*C. carpio*	China	MW675333 MW675334	Present study
*M.koi*	14–16	8–9	5–6	8–9	2.5-3	–	–	0.23	*C. carpio*	Japan	–	Kudo (1920)
*M.koi*	10–13	6–7	6	5–7	2–2.5	–	–	0.18	*C. carpio*	Japan	–	Nakai (1926)
*M.koi*	10.3–13.4	6.0–7.6	5.8–6.8	5.4–7.2	2.4–2.9	–	–	0.1–1	*C. carpio* *Carassius auratus* Linnaeus, 1758	Japan	–	Hoshina (1952)
*M. koi*	13–16	6–8	6.5	7–9	2–3	–	–	–	*C. carpio haematopterus* Temminck & Schlegel, 1846 *H. molitrix* Valenciennes, 1844	Russia Japan	–	Akhmerov (1960)
*M. koi*	14–16	7–9	5–6.7	7–9	–	–	–	0.25	*C. carpio haematopterus* *A. asmussi* Dybowski, 1872 *H. molitrix* *S. curriculus* Richardson, 1846	Russia Korea Japan	–	Shul’man (1966)
*M.koi*	14.1 ± 0.9 (11.7–16.1)	7.1 ± 0.6 (6.0–8.0)	6.6 ± 0.5 (5.1–7.9)	7.5 ± 0.7 (5.8–8.6)	3.0 ± 0.3 (2.4–3.6)	–	9	0.17 × 0.08	*C. carpio*	Britain	–	Crawshaw & Sweeting (1986)
*M.koi*	13.3 (12.5–15)	7.9 (7.0–9.0)	6.8 (6–8)	6.9 (6.7–7.4)	2.2 (2.0–2.7)	–	7–8	>1	*C. carpio*	Japan	–	Yokoyama et al. (1997)
*M.koi*	13.5 (12.0–15.0)	6.3 (5.0–7.5)	5.8 (5.0–6.5)	6.8 5.9–7.2	2.1 1.6–2.3	–	7–8	About 0.1			–	
*M.koi*	14.4 (13.2–15.6)	7.0 (6.6–7.8)	5.5 (4.8–6.2)	9.1 (8.4–9.6)	2.6 (2.4–3.0)	–	9–10	0.2–0.3	*C. carpio* *and others*	China	–	Chen & Ma (1998)
*M.koi*	15.4 (14.5–16.5)	8.3 (7.1–9.0)	–	10.1 (9.0–10.9)	3.1 (2.5–3.5)	–	9–11	–	*C. carpio*	USA	FJ841887	Camus & Griffin (2010)
*M.koi*	14.1 ± 0.5 (13.0–15.0)	7.0 ± 0.4 (6.0–8.0)	6.0 ± 0.2 (5.8–6.6)	7.5 ± 0.4 (7.0–8.3)	2.8 ± 0.2 (2.4–3.1)	–	9–10	0.6–2.3	*C. carpio*	China	FJ725077	Liu (2014)
*M.parakoi*	16.0 ± 0.8 (14.6–17.7)	7.8 ± 0.8 (6.7–9.8)	–	8.7 ± 0.5 (7.8–9.9)	3.0 ± 0.2 (2.6–3.6)	–	11–12	–	*C. carpio*	China	MH196558	Liu, Zhang & Zhao (2019)
*M.tanakai*	17.2 (15.4–18.6)	6.8 (6.3–8.4)	6.3 (5.9–6.8)	8.7 (7.6–9.4)	2.4 (2.0–2.7)	–	8–10	0.55 ×0.42	*C. carpio*	Japan	LC228235	Kato et al. (2017)
*M. orissae*	13.0–19.5	4.9–8.1	–	6.5–11.8	1.6–3.4	–	–	–	*Cirrhinus mrigala* Hamilton, 1822	India	–	Haldar, Samal & Mukhopadhyaya (1996)

**Notes.**

Abbreviations SL spore length SW spore width ST spore thickness PCL polar capsule length PCW polar capsule width PCT polar capsule thickness NPF number of polar filament coils *A* *Acanthorhodeus* *H* *Hypophthalmichthys* *S* *Squaliobarbus*

others include *A. chankaensis Dybowsky*,1872, *C. caspio haemtopterus* Temminck & Schlegel, 1846, *Carassius auratus*, *Carassius auratus auratus* Linnaeus, 1758, *Cirrhinus molitorella* Valenciennes, 1844, *Clarias batrachus* Linnaeus, 1758, *H. molitrix*, *Hypseleotris swinhonis* Günther, 1873, *S. curriculus*, *Sarcocheilichthys parvus* Nichols, 1930, *Senilabeo prochilus* Sauvage & Dabry de Thiersant, 1874 and *Varicorhinus simus* Sauvage & Dabry de Thiersant, 1874; -, data not available.

In the present study, some *Myxobolus* samples, which were collected from the gills of *C. carpio* in Chongqing and Guizhou and morphologically similar to *M. koi*, and their relationship with the related species including *M. koi* were comprehensively analyzed and defined as a new species *Myxobolus dajiangensis* n. sp.

## Materials & Methods

### New species

The electronic version of this article in Portable Document Format (PDF) will represent a published work according to the International Commission on Zoological Nomenclature (ICZN), and hence the new names contained in the electronic version are effectively published under that Code from the electronic edition alone. This published work and the nomenclatural acts it contains have been registered in ZooBank, the online registration system for the ICZN. The ZooBank LSIDs (Life Science Identifiers) can be resolved and the associated information viewed through any standard web browser by appending the LSID to the prefix http://zoobank.org/. The LSID for this publication is: D94613DA-E0C5-43A2-824B-9A6848A7C4C0. The online version of this work is archived and available from the following digital repositories: PeerJ, PubMed Central and CLOCKSS.

### Sample collection

Nine common carp, *C. carpio*, were collected from Chenjiaqiao Farmer’s Market, Shapingba District, Chongqing, and Golden Wharf Farmer’s Market, Bijiang District, Tongren, Guizhou, China, respectively. The fish measured between 11.6 cm to 15.9 cm in length. All fish were immediately transported to the laboratory for the parasitological examination. This study was approved by the Animal Care and Use Committee of the Key Laboratory of Animal Biology of Chongqing (Permit number: Zhao-20191010-01) and performed according to the recommendations in the Guide for the Care and Use of Animals at the Key Laboratory of Animal Biology of Chongqing, China.

### Fish examination and morphological identification

**Figure 1:**
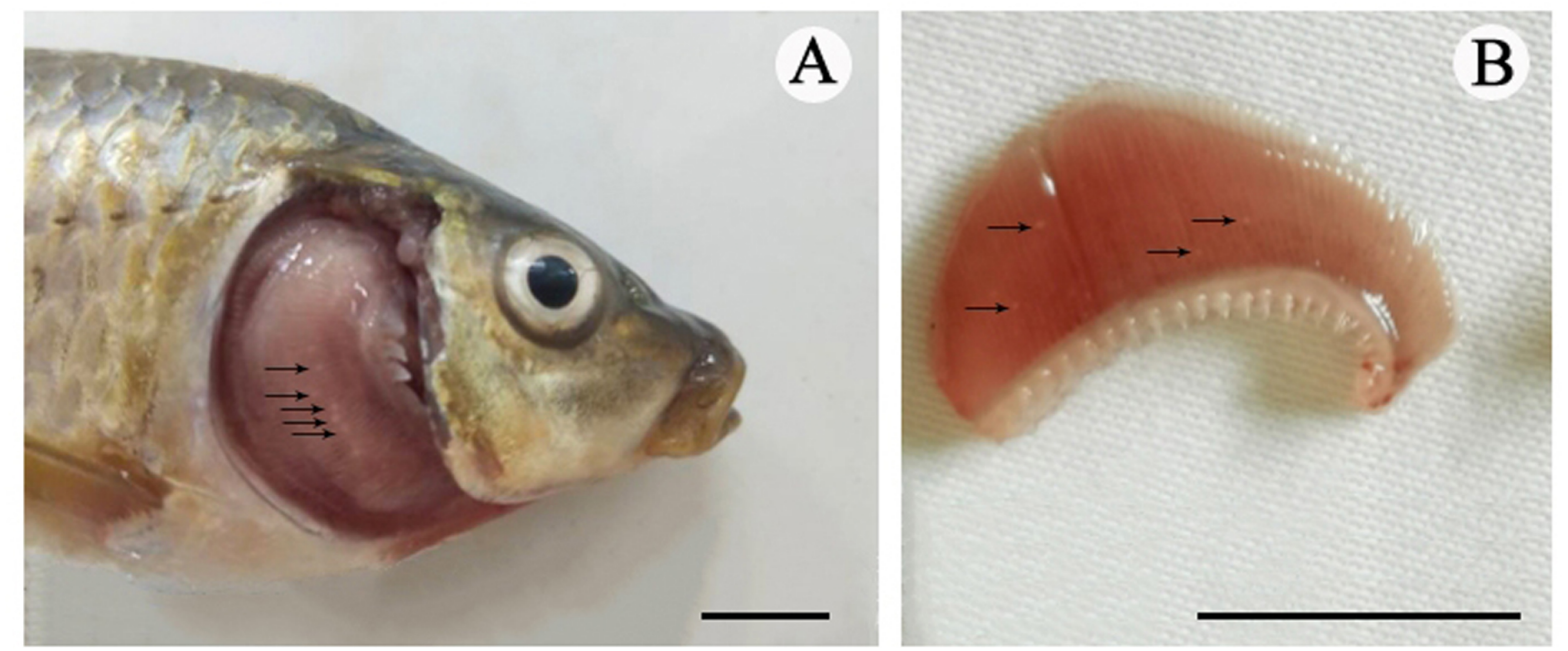
The cysts of *Myxobolus dajiangensis* n. sp. Arrows represent small white cysts of *Myxobolus dajiangensis* n. sp. in the gills of *C.carpio .* (A) The sample from Chongqing. (B) The sample from Guizhou. The bar is 1 cm.

The tissues and organs of the fish, including the gills, muscle, liver, intestine, and other organs were dissected. Samples were observed by the naked eyes and microscopy. Several small cysts were isolated from the infected gill lamellae (Fig. 1). The cysts were pierced with a fine needle to release the myxospores, which were then diluted with distilled water. The specimens were treated and identified as previously described (Zhao, Ma & Song, 2001). The morphological structure of the myxospores was observed, photographed, and determined using a Leica DM6000B microscope (Leica Microsystems, CMS GmbH, Germany) at 1,000× magnification. CorelDRAW 11 and Photoshop CS6 were used to illustrate our findings.

### Histological analysis

The infected gills were fixed in Bouin’s solution for 24 h, then dehydrated in a graded ethanol series, hyalinized in xylene, and embedded in paraffin wax. Tissue sections (5–6 µm) were stained by hematoxylin and eosin (H & E).

### DNA extraction, polymerase chain reaction (PCR), and sequencing

Genomic DNA of fresh myxospores from cysts was extracted using a DNeasy Blood & Tissue Kit (QIAGEN, Düsseldorf, Germany) following the manufacturer’s instructions. The SSU rDNA primers were as follows: 18E -5′CTG GTT GAT CCT GCC AGT-3′ (Hillis & Dixon, 1991) and 18R - 5′CTA CGG AAA CCT TGT TAC G-3′ (Whipps et al., 2003). PCR was performed using a Veriti™ 96-Well Thermal Cycler (Applied Biosystems, Singapore) in a 20 µL reaction system consisting of 7.2 µL dd H_2_O, 6 µL 2 × Taq Master Mix, 0.5 µL each primer, and 5.8 µL genomic DNA. Briefly, after an initial denaturation at 94 °C for 90 s, the amplifications were carried out with 35 cycles of 94 °C for 20 s, 58 °C for 20 s, and 72 °C for 2 min, followed by an extra extension at 72 °C for 5 min and held at 12 °C. 3 µL PCR amplicon was subjected to electrophoresis on 1% agarose gel stained with GoldView™ Nuclear Staining Dyes. PCR products were purified with the DNA Agarose Gel Extraction Kit (OMEGA Bio-Tek, Norcross City, GA, USA) and inserted into a pMD18-T vector (TAKARA, Otsu, Japan). The target nucleotide sequences were sent to the TSINGKE (Chongqing, China) for bidirectional sequencing.

### Molecular and phylogenetic analysis

The pairwise sequence similarities were calculated using BLASTn from GenBank. Multiple sequence alignments and divergences were carried out using MEGA6 with default parameters (Tamura et al., 2013). The predicted secondary structures, based on free energy minimization, were constructed for the selected species using RNA structure 5.2 with default settings. The obtained structures were displayed and manually adjusted using RNAViz 2.0 (Zhang et al. 2015). Three hypervariable regions of SSU rRNA (V4, V6 and V7) were selected to study their variability.

A total of 59 valid SSU rDNA sequences of *Myxobolus* species with over 94% similarity from Cyprinidae fishes in GenBank were selected and used for phylogenetic relationship analysis. *Kudoa quadricornis* (Whipps et al., 2003) and *Kudoa alliaria* Shulman et Kovaleva, 1979 were used as outgroups. A Bayesian inference (BI) tree was conducted using MrBayes 3.12 (Ronquist & Huelsenbeck, 2003). Maximum likelihood (ML) analyses were performed with RAxML HPC software (Stamatakis, Hoover & Rougemont, 2008) with 1,000 bootstrap replicates using the GTR + G model. Trees were initially examined in FigTree v1.4.2, then edited and annotated in Adobe Photoshop. The secondary structure model was available at the European ribosomal RNA database (Peer et al., 2000).

## Results

**Table:** 

**Figure 2:**
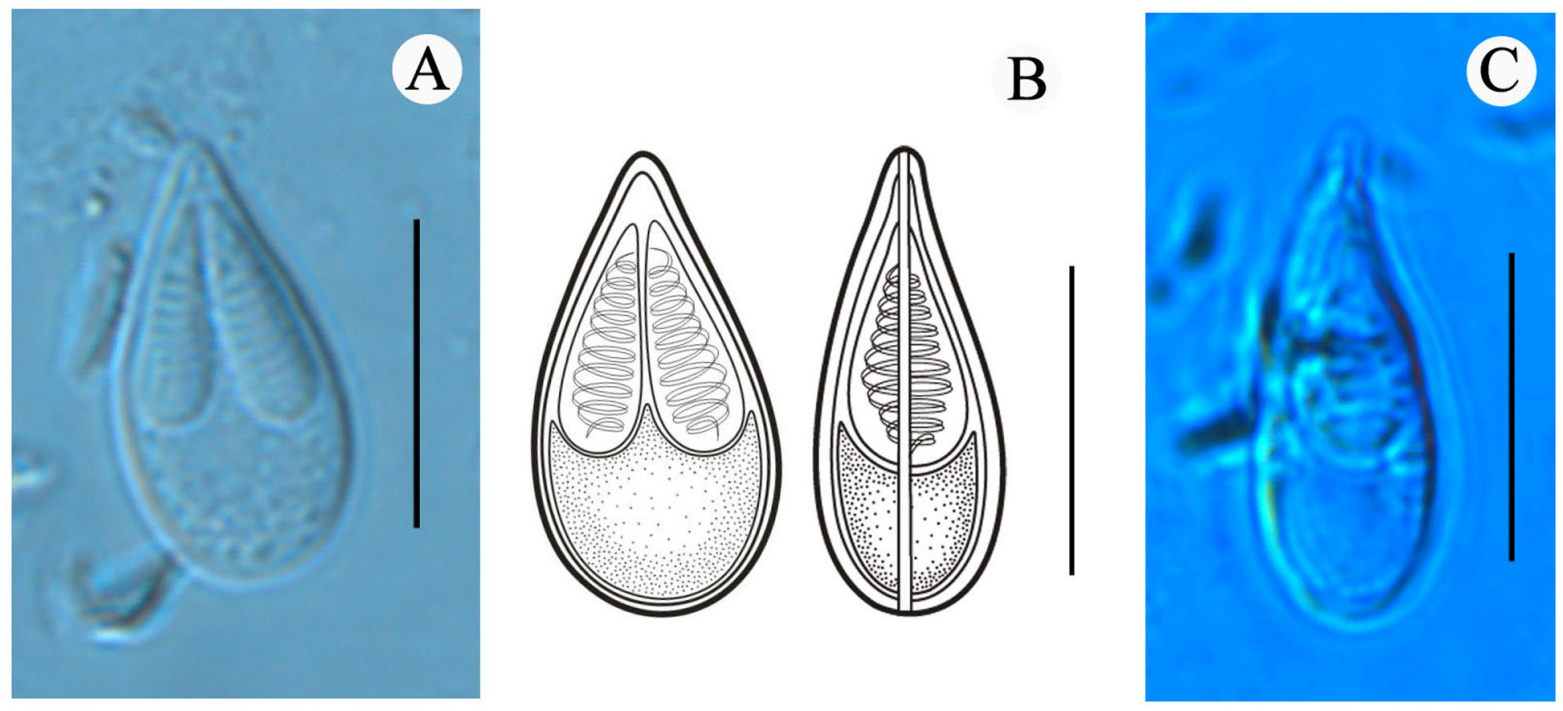
Mature spores of *Myxobolus dajiangensis* n. sp. collected from the gills of *C. carpio.*. (A) Mature spore in valvular view. (B) Schematic illustrations of *Myxobolus dajiangensis* n. sp. in frontal and sutural view. (C) Mature spore in sutural view. The bar is 10 µm.

*Myxobolus dajiangensis* n. sp. (Figs. 1–2)
Phylum: Cnidaria Hatschek, 1888
Class: Myxosporea Bütschli, 1881
Order: Bivalvulida Shulman, 1959
Family: Myxobolidae Thélohan, 1892
Genus: *Myxobolus* Bütschli, 1882

### Taxonomic summary

Type host: *Cyprinus carpio*

Site of infection: gill lamellae

Prevalence of infection: 100% (5/5) in Chongqing; 25% (1/4) in Guizhou.

Type locality: Chenjiaqiao, Shapingba District, Chongqing (29°30′N, 106°27′E), and Bijiang District, Tongren, Guizhou (27°71′N, 109°19′E), China.

Deposition of type materials: Syntype specimens (No. 202101) were deposited at the Collection Center of Type-specimens, Chongqing KLAB, Chongqing Normal University, China.

Etymology: The species was named after the main river flowing through the collection locality, Tongren City, Guizhou, China.

Vegetative stage: Many spherical whitish cysts, 0.2–0.8 mm in diameter, were located in the gill filaments (Fig. 1).

Morphological description: All the myxospores were mature. Vegetative spores were not observed in this study. The mature myxospores were elongated and pyriform in the frontal view, tapered towards an acuminate anterior end, rounded posterior end, and leptosomatic in the sutural view (Fig. 2). The myxospores were 14.8 ± 0.4 (13.9–15.6) µm in length, 8.0 ± 0.5 (7.3–9.1) µm in width (*n* = 60), and 5.5 µm in thickness (*n* = 1). The two polar capsules were slightly unequal in length. The length of the larger one was 8.0 ± 0.4 (7.1–8.8) µm, the smaller one was 7.4 ± 0.4 (6.1–8.0) µm, and the width of both polar capsules was 2.5 ± 0.2 (2.0–3.2) µm (*n* = 60). Polar filaments were coiled with approximately 10 turns (ranging from 9 to 11) and perpendicularly situated to the longitudinal axis of the polar capsule. The aspect ratio of the myxospore was 1:0.5, and the aspect ratio of the polar capsule was 1:0.3. Moreover, the ratio of myxospore length to polar capsule length was 1:0.5. The ratio of the myxospore width to polar capsule width was 1:0.3. The structures, including the intercapsular appendix, mucous envelope, iodinophilous vacuole and sutural edge marking were not observed (Fig. 2).

### Histology

**Figure 3:**
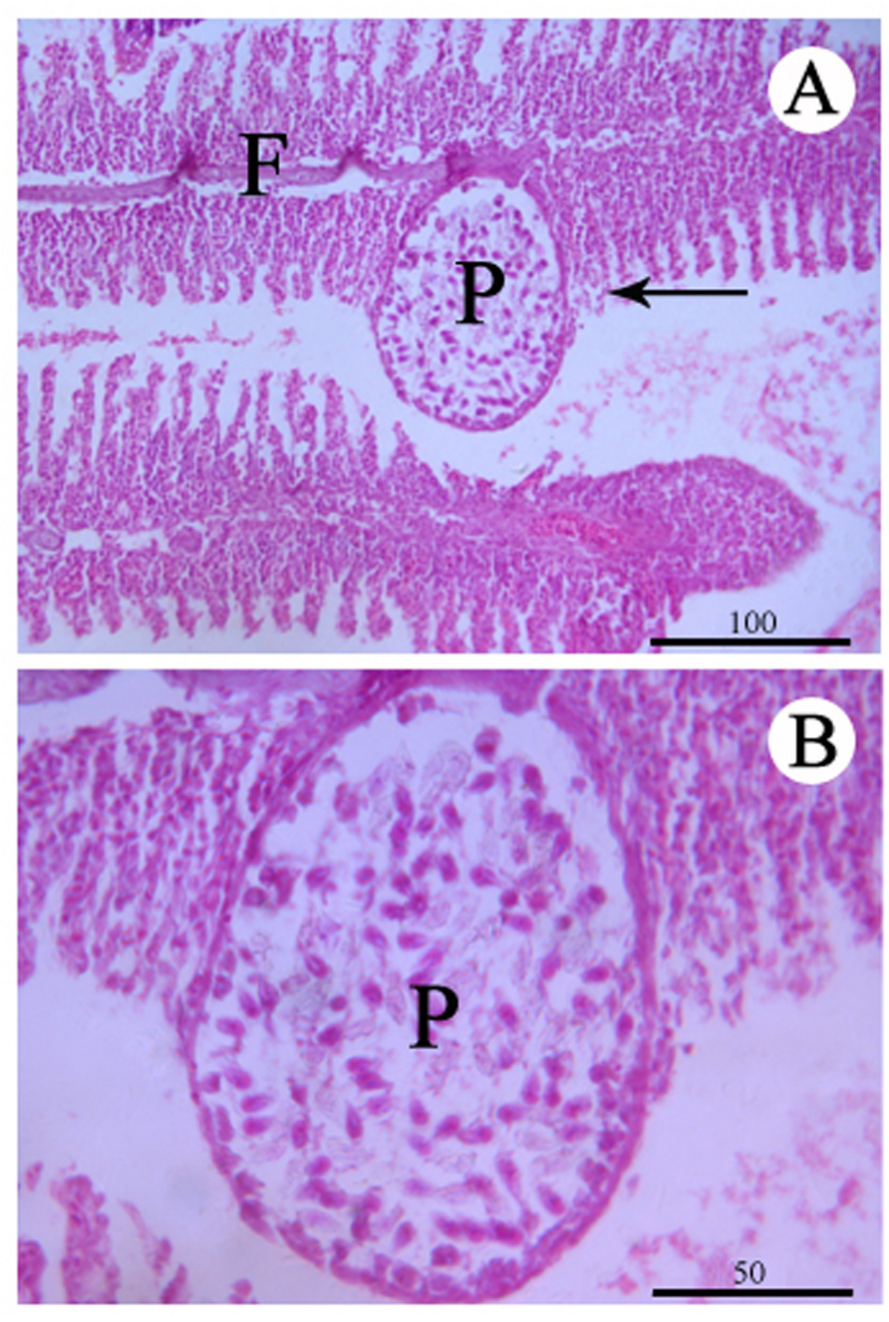
Histological section of the infected gill stained with Hematoxylin & Eosin. (A) Plasmodium (P) of *Myxobolus dajiangensis* n. sp. shows the interlamellar-vascular type and involves in the neighboring gill lamellae in *C. carpio*. (B) An enlargement of (A). F, gill filament.

No remarkable external clinicopathological features were observed for the examined fish. Host responses to the infection led gill lamellae to expand to accommodate the plasmodia (an outer tissue consisting of epithelium cells and pillar cells). An abundance of plasmodia was observed in the secondary lamellae and a large plasmodium in the gill lamellae pushed and deformed the gill lamellae on both sides (Figs. 1 and 3). Any pseudoeosinophil cells and distinct inflammatory responses were not observed at the infection sites.

### Molecular and phylogenetic analysis

Two SSU rDNA sequences with 1,885 nt and 1,969 nt were successfully obtained from Chongqing and Guizhou, respectively. These sequences were deposited in GenBank, and Table 1 lists the accession numbers. The alignment results indicated that the present species had a 100% similarity with *M. koi* (FJ710800), which was also derived from the gills of *C. carpio*. There was a similarity of less than 98% with other myxosporeans, including a 97.76% similarity with *M. tanakai* (LC228236), a 97.37% similarity with *M. parakoi* (MH196558), a 97.33% similarity with *M. koi* (MH196560), a 97.32% similarity with *M. koi* (FJ841887), a 97.03% similarity with *M. koi* (FJ725077), and a 96.87% similarity with *M. koi* (KT240127).

**Figure 4:**
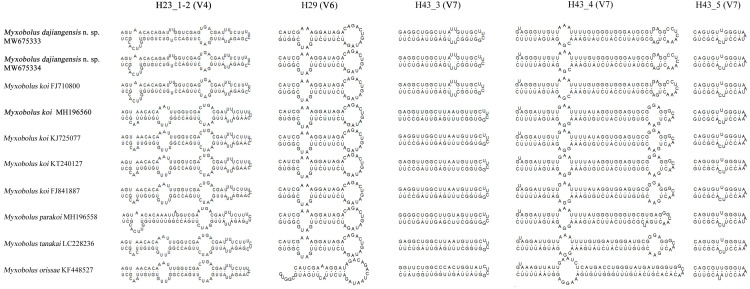
The secondary structures of SSU rRNA V4, V6 and V7 of *Myxobolus dajiangensis* n. sp. and similar species in morphology. Items in bold represent the SSU rRNA secondary structures of *Myxobolus dajiangensis* n. sp. and *M. koi* submitted by our research team in the present study

Compared with other species similar in morphology, the secondary structures of SSU rRNA of our specimens coincided with those of *M. koi* (FJ710800). However, they were not the same as those of SSU rRNA of *M. koi* parasites (MH196560, FJ841887, KT240127, and KJ725077), *M. parakoi*, *M. tanakai* and *Myxbolus orissae* (Haldar, Samal & Mukhopadhyaya, 1996) (Fig. 4). The left internal bulges of H23_1-2 in the V4 of the present species were the same as those of *M. parakoi*, but bigger than those of *M. koi* parasites (MH196560, FJ841887, KT240127, and KJ725077), *M. tanakai* and *M. orissae*. The second internal bulges of H23_1-2 in the present species were smaller than those of *M. koi* parasites (MH196560, FJ841887, KT240127, and KJ725077), *M. tanakai*, and *M. orissae*, except for *M. parakoi*. The third internal bulges of H23_1-2 in the studied species and *M. koi* (FJ710800) were smaller than those of other similar species in morphology. The lateral bulges of H23_1-2 showed no difference with these of *M. parakoi* and *M. tanakai* but differed from those of *M. koi* parasites (MH196560, FJ841887, KT240127, and KJ725077) and *M. orissae*. There was an internal bulge in H43_3 in the V7 of the present species and *M. koi* (FJ710800) but not in others. The biggest difference of H43_4 in the V7 between the present species and other similar species was the right internal bulge and a hairpin loop. H29 in the V6 and H43_5 in the V7 of the present species were identical with those of other species except for *M. orissae*.

**Figure 5:**
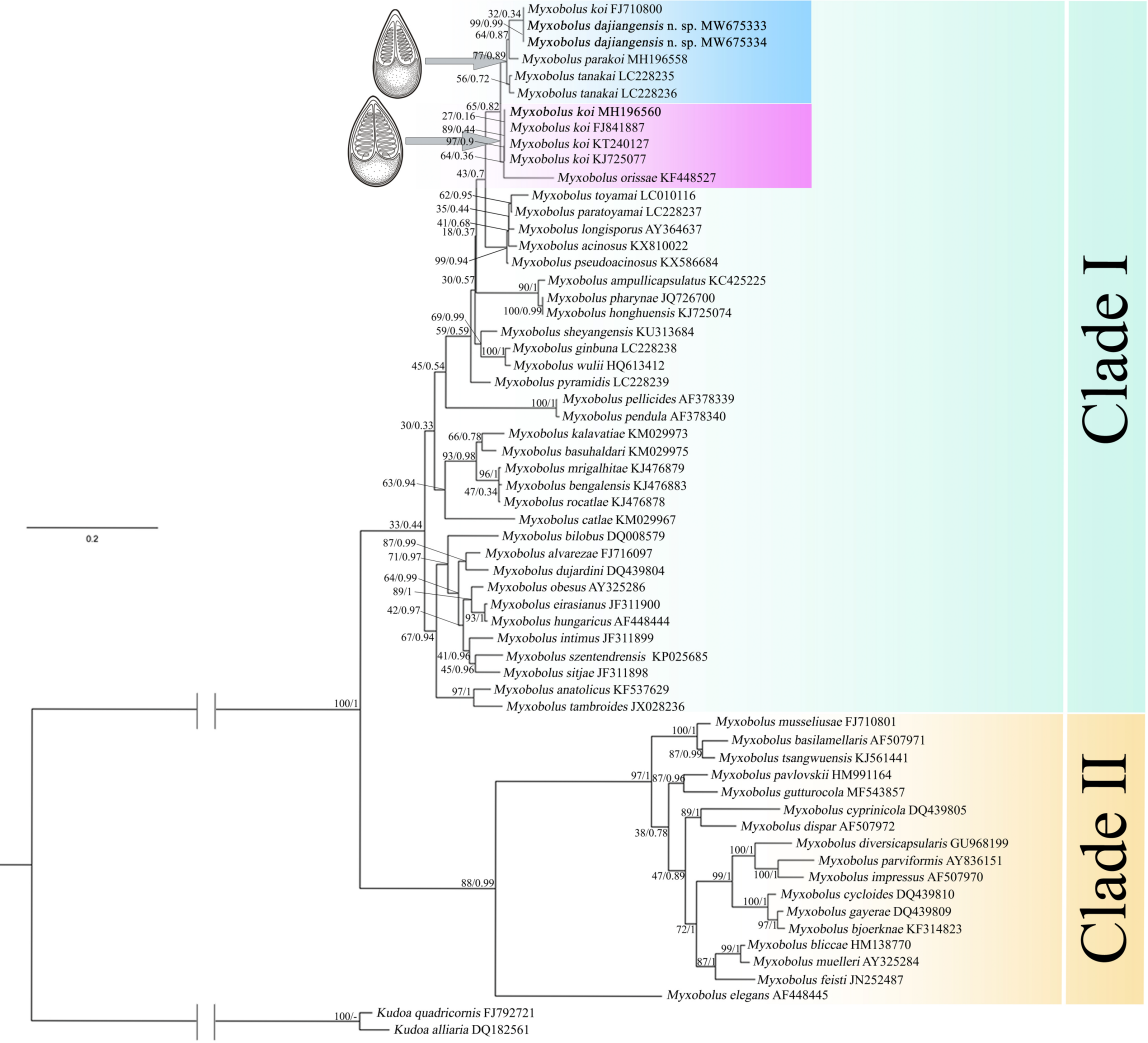
ML and BI phylogenetic tree based on SSU rDNA sequences of *Myxobolus dajiangensis* n. sp. and its closely related *Myxobolus* species. Numbers given at nodes of branches are bootstrap support (BS) and Bayesian posterior probabilities (PP). The species in the present study is indicated in bold. //, shortened to one-third of the original length.

In the present study, the phylogenetic results of ML and BI showed highly similar topology, which were incorporated into one tree based on the ML tree (Fig. 5). The myxospore morphology in this phylogenetic tree was closely correlated to their phylogenetic relationships. The species with pyriform spores sharing a tapering anterior end were clustered together in Clade I, and those possessing ellipsoidal spores with blunt anterior ends were clustered in Clade II. The two sequences from Guizhou and Chongqing in this study and *M. koi* (FJ710800) were first clustered together, forming a sister relationship with *M. parakoi*, and joined with *M. tanakai* to form a small subclade. The other *M. koi* parasites (MH196560, FJ841887, KT240127, and KJ725077) branched independently and formed a sister relationship with *M. orissae* to compose another small subclade. Then the above mentioned two subclades were clustered together in Clade I.

## Discussion

A holistic approach integrating molecular data, phenotypic features, tissue tropism and host specificity to identify and describe species has been widely accepted (Zhao et al., 2013; Fiala, Bartošová-Sojková & Whipps, 2015; Liu, Yang & Zhao, 2016; Liu, Zhang & Zhao, 2019). Morphologically, although the mature myxospores of the present species closely resembles *M. koi*, *M. tanakai*, *M. orissae*, and *M. parakoi*, which all parasitize in *C. carpio* except for *M. orissae*, the morphological structural features are inconsistent more or less. The present species resembles *M. koi* with their elongated pyriform spores but has a narrower front end compared with the original *M. koi* reported by Kudo (1920) and described later by Camus & Griffin (2010). Moreover, unlike the *M. koi* originally reported by Kudo (1920) and Camus & Griffin (2010), the polar capsules of the present species are full of the myxospore cavity in the sutural view (Fig. 2). The myxospore of the present species is significantly shorter compared with *M. tanakai* (Table 1). The two polar capsules of the present species also differs in length compared with corresponding polar capsules of *M. parakoi* (Table 1). In addition to the morphometric differences between the present species and *M. orissae* as well as their hosts are also various (Table 1). Therefore, the present species is considered as a novel species in morphology and host. Molecularly, several reports have indicated that intraspecies molecular criteria are less than 1% (Molnár et al., 2012). In the present study, the SSU rDNA divergence is 0.000 between *M. koi* (FJ710800) and the present species. The secondary structures in the V4, V7, and V6 regions of the SSU rRNA of *M. koi* (FJ710800) and the present species are also identical. All of these suggest that *M. koi* (FJ710800) should be attributed to the present species, while not *M. koi* although there is no its morphological data reported. However, divergences of SSU rDNA gene between the present species and other closely related species, namely *M. tanakai* (LC228235, LC228236), *M. parakoi* (MH196558) and *M. koi* (MH196560, FJ841887, KT240127, and KJ725077), range from 2.2% to 2.7%, which fall out of the intraspecific divergent criteria. As for the secondary structures in the V4, V7, and V6 regions of the SSU rRNA, the present species is remarkably different those of the closely related species (Fig. 5). Phylogenetically, *M. koi* (FJ710800) and the present species group independently and have no sister relationship with other sequences of *M. koi*, which further proves that *M. koi* (FJ710800) and the present species are the congener and different organisms with *M. koi* (Fig. 5). In addition, the gathering pattern of the present species and the related species in the phylogenetic tree is consistent with that of their myxospore shapes. In a word, morphological and molecular data as well as phylogenetic result all indicate that the present species was different from the other closely related species and recognized as a new species, named *Myxobolus dajiangensis* n. sp. *M. koi* (FJ710800) is misidentified and the congener with the present species. Also, our analyses of the secondary structure indicates that V4 H23_1-2 and V7 H43_4 are valid markers and can be used as barcoding to identify these morphologically similar *Myxobolus* species with *Myxobolus dajiangensis* n. sp.

Histologically, the present species can be designated as intralamellar-vascular type according to the guidelines proposed by Molnár (2002). Its intralamellar location seems to be one of the most common types of plasmodium development. There are many myxosporeans of this type, such as *M. koi* with the small-cysts type, *Myxobolus macrocapsularis* Reuss, 1906 and *Myxobolus muellericus* Molnár, 2006 (Yokoyama et al., 1997; Molnár, 2002, Molnár et al., 2006, Molnár, Cech & Székely, 2011). Our result has shown that a myxobolid infection structurally destroys the gill lamellae by the plasmodia. Therefore, *M. dajiangensis* n. sp. might destroy the functional respiratory surface of the gills if the infection was serious enough (Abdel-Baki et al., 2014).

In summary, *Myxobolus dajiangensis* n. sp. is a new species, based on the morphological and molecular data. This study provides the foundational data for figuring out the cryptic species of *M. koi.* Histologically, *Myxobolus dajiangensis* n. sp. is a potential threat to its host.

## Supplemental Information

10.7717/peerj.13023/supp-1 Supplemental Information 1 BI phylogenetic tree based on SSU rDNA sequences of *Myxobolus dajiangensis* n. sp. and its closely related *Myxobolus* species

10.7717/peerj.13023/supp-2 Supplemental Information 2 ML phylogenetic tree based on SSU rDNA sequences of *Myxobolus dajiangensis* n. sp. and its closely related *Myxobolus* species

10.7717/peerj.13023/supp-3 Supplemental Information 3 Raw morphometic data of *Myxobolus dajiangensis*

10.7717/peerj.13023/supp-4 Supplemental Information 4 18S rDNA sequences of *Myxobolus dajiangensis* n. sp

## Additional Information and Declarations

### DNA Deposition

The following information was supplied regarding the deposition of DNA sequences: The sequences are available at GenBank: MW675333 and MW675334.

### Data Availability

The following information was supplied regarding data availability: The raw measurements, BI tree and ML tree are available in the Supplementary Files.

### New Species Registration

The following information was supplied regarding the registration of a newly described species: Publication LSID: urn:lsid:zoobank.org:pub:D94613DA-E0C5-43A2-824B-9A6848A 7C4C0 *Myxobolus dajiangensis* n. sp. LSID: urn:lsid:zoobank.org:act:0B2B80F6-E984-4558-B48F-A33675D4FEA0
