# Supplementary figures and images for: Morphological description and molecular identification of Myxobolus dajiangensis n. sp. (Myxozoa: Myxobolidae) from the gill of Cyprinus carpio in southwest China
Source: PeerJ. 2022 Mar 4;10:e13023. doi: 10.7717/peerj.13023 (PMC8900608; doi:10.7717/peerj.13023)

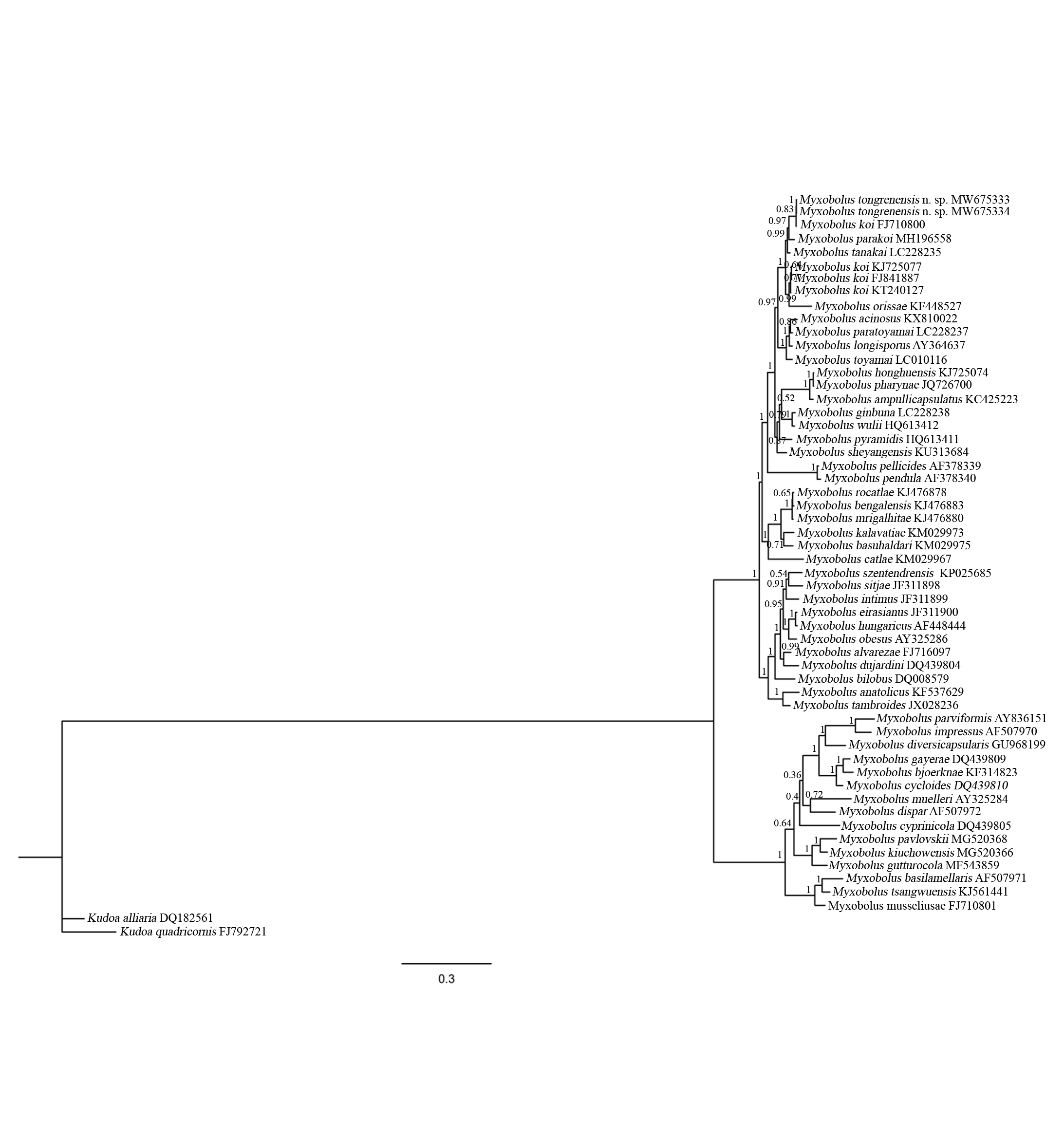

Supplement: Supplemental Information 1 [file peerj-10-13023-s001.jpg]
